# Oligodendrocytes Prune Axons Containing α-Synuclein Aggregates In Vivo: Lewy Neurites as Precursors of Glial Cytoplasmic Inclusions in Multiple System Atrophy?

**DOI:** 10.3390/biom13020269

**Published:** 2023-02-01

**Authors:** Francesco De Nuccio, Marianna Kashyrina, Francesca Serinelli, Florent Laferrière, Dario Domenico Lofrumento, Francesca De Giorgi, François Ichas

**Affiliations:** 1 Department of Biological and Environmental Sciences and Technologies, Section of Human Anatomy, University of Salento, I-73100 Lecce, Italy; 2 Institut des Maladies Neurodégénératives, CNRS, Université de Bordeaux, UMR 5293, 33076 Bordeaux, France

**Keywords:** α-Synuclein, PFFs, seeding, spread, anterior commissure, Lewy neurite, glial cytoplasmic inclusion, oligodendrocyte, axonal pruning, multiple system atrophy

## Abstract

α-Synucleinopathies are spreading neurodegenerative disorders characterized by the intracellular accumulation of insoluble aggregates populated by α-Synuclein (α-Syn) fibrils. In Parkinson’s disease (PD) and dementia with Lewy bodies, intraneuronal α-Syn aggregates are referred to as Lewy bodies in the somata and as Lewy neurites in the neuronal processes. In multiple system atrophy (MSA) α-Syn aggregates are also found within mature oligodendrocytes (OLs) where they form Glial Cytoplasmic Inclusions (GCIs). However, the origin of GCIs remains enigmatic: (i) mature OLs do not express α-Syn, precluding the seeding and the buildup of inclusions and (ii) the artificial overexpression of α-Syn in OLs of transgenic mice results in a burden of soluble phosphorylated α-Syn but fails to form α-Syn fibrils. In contrast, mass spectrometry of α-Syn fibrillar aggregates from MSA patients points to the neuronal origin of the proteins intimately associated with the fibrils within the GCIs. This suggests that GCIs are preassembled in neurons and only secondarily incorporated into OLs. Interestingly, we recently isolated a synthetic human α-Syn fibril strain (1B fibrils) capable of seeding a type of neuronal inclusion observed early and specifically during MSA. Our goal was thus to investigate whether the neuronal α-Syn pathology seeded by 1B fibrils could eventually be transmitted to OLs to form GCIs in vivo. After confirming that mature OLs did not express α-Syn to detectable levels in the adult mouse brain, a series of mice received unilateral intra-striatal injections of 1B fibrils. The resulting α-Syn pathology was visualized using phospho-S129 α-Syn immunoreactivity (pSyn). We found that even though 1B fibrils were injected unilaterally, many pSyn-positive neuronal somas were present in layer V of the contralateral perirhinal cortex after 6 weeks. This suggested a fast retrograde spread of the pathology along the axons of crossing cortico-striatal neurons. We thus scrutinized the posterior limb of the anterior commissure, i.e., the myelinated interhemispheric tract containing the axons of these neurons: we indeed observed numerous pSyn-positive linear Lewy Neurites oriented parallel to the commissural axis, corresponding to axonal segments filled with aggregated α-Syn, with no obvious signs of OL α-Syn pathology at this stage. After 6 months however, the commissural Lewy neurites were no longer parallel but fragmented, curled up, sometimes squeezed in-between two consecutive OLs in interfascicular strands, or even engulfed inside OL perikarya, thus forming GCIs. We conclude that the 1B fibril strain can rapidly induce an α-Syn pathology typical of MSA in mice, in which the appearance of GCIs results from the pruning of diseased axonal segments containing aggregated α-Syn.

## 1. Introduction

As a result of the conversion of α-Synuclein (α-Syn) protein monomers into prion-like fibril assemblies capable of replicating and spreading into the brain (reviewed in [1]), several neurodegenerative diseases are classified as α-synucleinopathies, such as Parkinson’s disease (PD), dementia with Lewy bodies (DLB), and multiple system atrophy (MSA) [2,3,4]. From a pathological point of view, both PD and DLB are characterized by the presence of α-Syn fibrils populating intraneuronal inclusions referred to as Lewy bodies (LBs) in the perikarya and as Lewy neurites in the processes [3]. While PD manifests primarily as movement disorders due to the early and extensive loss of dopaminergic neurons in the substantia nigra pars compacta, clinical diagnosis of DLB is made when a patient develops dementia within one year of onset of motor symptoms of PD [5]. On the other hand, MSA has clinical and pathological characteristics that differ from those of PD and DLB. MSA patients mainly show parkinsonism, cerebellar ataxia, and autonomic failure; their condition declines rapidly and survival is shorter than PD and DLB [6]. MSA is characterized by abundant oligodendroglial α-Syn aggregates, referred to as Glial Cytoplasmic Inclusions (GCIs), taking precedence over early Neuronal Intranuclear Inclusions (NIIs), Neuronal Cytoplasmic Inclusions (NCIs), and Lewy neurites (LNs) [6,7,8] (for a review see [9]). α-Syn, which is highly expressed by neurons and rarely by glia, is thought to spontaneously form amyloid fibrils when it accumulates under conditions of overexpression or defective degradation [10,11]. In this context, several lines of α-Syn transgenic mice driven by oligodendrocyte-specific promoters were generated in order to model MSA in mice [12,13,14]. However, these mice not only exhibit very mild motor disturbance phenotypes compared to MSA [15], but the massive “GCI-like” burden of oligodendroglial α-Syn forming in their brains—although phosphorylated—is also devoid of α-Syn fibrils [16]. This casts serious doubts on the ability of these animal models to replicate the pathological mechanisms of MSA especially since there is neither evidence of elevation in α-Syn expression in the brain regions harboring GCIs or in oligodendrocytes (OLs) in patients with MSA [17,18,19]. Thus, the pathological mechanisms of GCI formation in the genesis of MSA remain unknown.

## 2. Materials and Methods

### 2.1. α-Syn Expression

Escherichia coli strain BL21(DE3) plysS was transformed with pET24–α-Syn vector by electroporation and plated onto a Luria broth agar plate containing kanamycin (30 μg/mL). A preculture in 5 mL of Luria broth medium was inoculated with one clone and incubated at 37 °C under 200 rpm shaking for 4 h. The expression on α-Syn was carried out in M9 minimal medium containing 13C glucose (2 g/L) and 15NH4Cl (1 g/L) as carbon and nitrogen sources. Cells from the Luria broth preculture were recovered by centrifugation (1000× *g*, 10 min) and used for inoculating 200 mL of M9 medium. Cells were grown overnight at 37 °C under 200 rpm shaking and then diluted in 2 L of culture. Protein expression was induced by adding 1 mM isopropyl-β-d-thiogalactopyranoside during the exponential phase, evaluated at an optical density at 600 nm reaching 0.8. Cells were harvested after 4 to 5 h of culture at 37 °C by 6000× *g* centrifugation (JLA 8.1000, Beckman Coulter, Villepinte, France), and pellet was kept at −20 °C before purification.

### 2.2. α-Syn Purification

The pellets were resuspended in lysis buffer (10 mM Tris and 1 mM EDTA (pH 7.2) with protease inhibitor tablets (cOmplete, EDTA-free protease inhibitor cocktail, Roche, Basel, Switzerland)) and sonicated at 50% max energy, 30 s on and 30 s off for three rounds with a probe sonicator (Q-Sonica, Newtown, CT, USA). The sonicated pellets were centrifuged at 20,000× *g* for 30 min, and the supernatant was saved. The pH of the supernatant was then reduced to pH 3.5 using HCl, and the mixture stirred at room temperature (RT) for 20 min and then centrifuged at 60,000× *g* for 30 min. The pellets were discarded. The pH of the supernatant was then increased to pH 7.4 with NaOH and then dialyzed against 20 mM tris-HCl (pH 7.40) and 100 mM NaCl buffer before loading onto a 75 pg HiLoad 26/600 Superdex column equilibrated with the same buffer with ÄKTA pure system. Monomeric fractions were collected and concentrated if needed by using Vivaspin 15R 2 kDa cutoff concentrator (Sartorius Stedim, Göttingen, Germany). Purification fractions were checked by using polyacrylamide gel electrophoresis (PAGE) tris-tricine 13% dying with ProBlue Safe Stain. Protein concentration was evaluated spectrophotometrically by using absorbance at 280 nm and an extinction coefficient of 5960 M^−1^ cm^−1^.

### 2.3. α-Syn Fibrillization

Solutions of monomeric α-Syn at 4 to 5 mg/mL in saline (H_2_O, 100 mM NaCl, and 20 mM tris-HCl (pH 7.40)) were sterilized by filtration through 0.22 μm Millipore single-use filters and stored in sterile 15 mL conical falcon tubes at 4 °C. Sterilized stock was then distributed into safe-lock Biopur individually sterile-packaged 1.5 mL Eppendorf tubes as 500 μL aliquots and were seeded with 1% of the α-Syn 1B fibrils described in [20]. The tubes were cap-locked and additionally sealed with parafilm. All previous steps were performed aseptically in a particle-free environment under a microbiological safety laminar flow hood. The samples were loaded in a ThermoMixer (Eppendorf, Hamburg, Germany) in a 24-position 1.5 mL Eppendorf tube holder equipped with a heating lid. Temperature was set to 37 °C, and continuous shaking at 2000 rpm proceeded for 4 days.

### 2.4. Sonication

Prior to intracerebral injections, 1B α-Syn fibril stocks (4 mg/mL) were distributed in cap-locked, sterile 0.5 mL polymerase chain reaction (PCR) tubes (Thermo Fisher Scientific, Bordeaux, France). Sonication was performed at 25 °C in a Bioruptor Plus water bath sonicator (Diagenode, Liège, Belgium) equipped with thermostatic control and automated tube carousel rotator. The sonication power was set to “high”, and 10 cycles of 30 s on followed by 10 s off were applied.

### 2.5. In Vivo α-Syn Pathology and Histology

Adult male 129SV mice were housed in a temperature-controlled (22 °C) and light-controlled environment on a 12 h light/12 h dark cycle with access to food and water ad libitum. Male mice were selected to avoid gender bias in additional olfaction-dependent behavioral analyses (not shown here). The study design was approved by the French Ministry of Research and all experimental procedures were conducted in accordance with the European Communities Council Directive (2010/63/EU) for care of laboratory animals. The mice (6–8 weeks old) unilaterally received 2 μL of sonicated α-Syn fibrils 1B (4 mg/mL) by stereotactic delivery at a flow rate of 0.4 μL/min, and the pipette was left in place for 5 min after injection to avoid leakage. Delivery was performed within the right striatum (AP, −0.1; L, +2.5; DV, +3.8), n = 6 mice plus 2 controls. Animals were euthanized after 6 weeks (n = 3 plus 3 solvent-injected controls) or 6 months (n = 3 plus 3 solvent-injected controls) and were transcardially perfused with tris-buffered saline (pH = 7.4) followed by 4% paraformaldehyde in PBS pH = 7.4 at 4 °C. Brains were subsequently postfixed in the same fixative, paraffin embedded, and 10 µm sections were obtained with a rotative microtome (Leica, Milan, Italy). The sections of interest were deparaffinized and processed for epitope retrieval: the slides were immersed in citrate buffer pH 6 (Dako Agilent Technologies, Les Ulis, France) and placed in a pressure cooker (Bio SB, Santa Barbara, CA, USA) at 120 °C for 10 min. After a cooling period of 20 min, the slides were washed twice for 5 min in PBS at room temperature. They were then processed for simple or double immunofluorescence using the following primary antibodies diluted at 1/500, or their combinations: EP1536Y (Abcam) or pSyn#64 (Wako) for detecting phospho-S129–positive α-Syn amyloid aggregates; D37A6 (Cell Signaling) for detecting mouse α-Syn; Neurofilament (NF-200, Sigma Aldrich, St. Quentin-Fallavier, France, N4142) for detecting commissural axons [21]; Olig-2 (Abcam, rabbit monoclonal; Millipore, 211F1.11 clone, mouse monoclonal) for detecting OL nuclei, CNPase (Abcam) for detecting myelin sheaths; T46 (anti Tau, Thermofisher, Bordeaux, France) and mouse anti-QKI7 (Clone N183/15) (UC Davis/NIH NeuroMab Facility, Davis, CA, USA) for detecting the cytoplasm of mature OLs in the anterior commissure [22,23,24]; EP1532Y (anti-Tyrosine Hydroxylase, Abcam) for detecting the nigrostriatal tract; anti GFAP (Sigma) for detecting mature astrocytes. Draq7 was used to image the nuclei. The AlexaFluor-coupled secondary antibodies were from Thermo Fisher (Alexa 488, 568, and 674). The sections were acquired using a Pannoramic slide scanner (3D HISTECH, MM France) in epifluorescence mode, and multichannel fluorescence optical sections of the samples were performed (thickness < 0.8 µm) using a Leica SP5 Laser Scanning Confocal Microscope equipped with a spectral detector, 488, 561, and 633 nm laser lines, a motorized X-Y stage, and a mixed stepping motor/piezo Z controller. The objective was 40×, and Z step size was set to 0.5 µm to produce stacks of 15 to 20 Z planes. Pinhole was set to 1 airy unit. For 3D reconstructions and volume rendering/animations (corresponding to 360° tilt series of composite max pixel projections images), raw 3-channel Z-stack images were processed offline using Icy 2.3 [25].

## 3. Results

A current hypothesis to explain MSA pathology is that fibrils from an MSA-specific α-Syn strain could specifically gain access to the OL cytoplasm and seed the formation of GCIs [26] (recently reviewed in [27]). However, this point of view is weakened by the fact that normal OLs cultured to a mature, myelin-competent state are devoid of α-Syn [28], i.e., they are deprived of a soluble substratum capable of being converted into an inclusion by a seed. In addition, no increase in α-Syn expression that could render OLs “seeding-responsive” has been observed during MSA [17,18,19]. Thus, as starting point of our in vivo study in mice, we first explored α-Syn in OLs in situ. Our observations confirmed that mature OLs lack α-Syn in vivo. 

**Figure 1:**
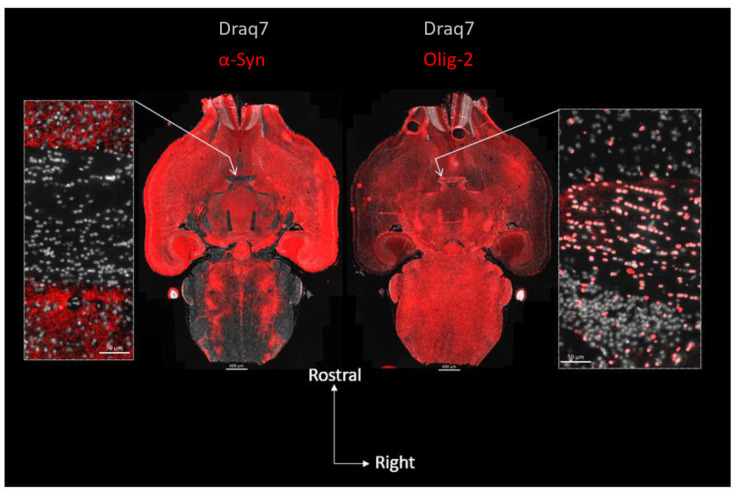
Topography of α-Syn expression and OL distribution in the mouse brain: absence of α-Syn in mature commissural OLs. Center: two consecutive full brain horizontal sections (10 µm) in which α-Syn (left, red; D37A6 antibody) or the OL nuclear marker Olig-2 (right, red) were revealed using immunofluorescence and imaged using epifluorescence slide scanning. Lateral images are close-up views of the anterior commissure regions pointed at by arrows. Nuclei were revealed with Draq7 (white).

**Figure 2:**
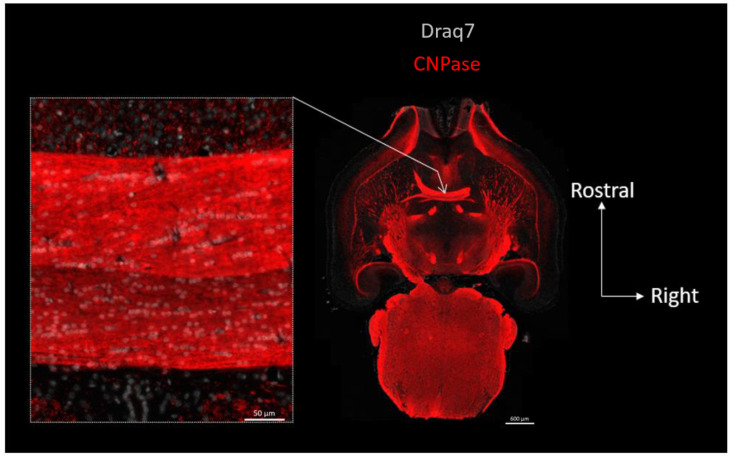
Topography of CNPase expression in the mouse brain: OLs residing in the anterior commissure are myelinating this interhemispheric fiber tract. Right: full brain horizontal section in which the myelin and OL marker CNPase (red) was revealed using immunofluorescence and imaged using epifluorescence slide scanning. Left image is a close-up view of the anterior commissure region pointed at by arrow. Nuclei were revealed with Draq7 (white).

In Figure 1, we revealed mouse α-Syn or the OLs in consecutive whole brain semi-horizontal sections using, respectively, a rodent-specific anti-α-Syn antibody (D37A6) or the OL marker Olig-2. The sections, which encompass the full rostro-caudal span of the nigrostriatal pathway, make it clear that there is an inverse relationship between the levels of α-Syn and the density of OLs. Consistent with its localization in axon terminals, α-Syn which is at its highest in the gray matter of the telencephalon, but also in the substantia nigra pars reticulata, vanishes in the white matter of the pons and of the brainstem, to completely disappear in major myelinated axonal tracts, such as for instance, the anterior commissure (Figure 1, zoomed views). The vast majority of cell bodies present in the latter tract revealed by their stained nuclei (Draq7) correspond to Olig-2-positive interfascicular OLs often organized in typical rows (see Olig-2 zoomed view on the right) and show no expression of α-Syn (see α-Syn zoomed view on the left). See also Supplementary Figure S1. Examination of the myelin sheaths in a third section (using an anti-CNPase antibody) shows that the α-Syn-negative commissural OLs are extensively myelinating the interhemispheric axons constituting this tract (Figure 2). Note that both the anterior and posterior nerves of the anterior commissure are easily evidenced here.

In the absence of α-Syn expression in OLs, an alternative possibility to explain the appearance of α-Syn aggregates in these cells is that the GCIs observed in MSA are “imported aggregates” that are first seeded, assembled and compacted in diseased neurons, and only secondarily passed over to OLs [29]. This view is supported by the fact that most of the proteins found firmly associated with the insoluble α-Syn fibrils in MSA are specifically neuronal and similar to those found in PD aggregates [29]. The hypothesis of a neuron-to-OL aggregate transfer is further supported by the key observations made by the late J. Q. Trojanowski and his colleagues [30] who described the delayed formation of GCIs in mice intracerebrally injected with homologous mouse α-Syn fibrils, also referred to as mouse “PFFs” (standing for “Pre-Formed α-Syn Fibrils”), a delay leaving room to the buildup and decay of a neuronal α-Syn pathology “primer”. 

The fact that mouse PFFs could cause the appearance of GCIs in this latter work particularly caught our attention. Indeed, a property of mouse PFFs is that they are poorly stained by the amyloid probe Thioflavin T (ThT) [31] like the case for the α-Syn fibril strains related to MSA [26,32,33,34] (see Table 1). Since 1B fibrils belong to this latter group and seed neuronal intranuclear inclusions (NIIs) [34] that are typical of MSA [35], we investigated whether they could also cause the delayed appearance of GCIs in OLs in vivo.

Six weeks after the intracerebral injection of human 1B fibrils into the right striatum of wild-type mice, we observed the seeding and the spread of an α-Syn pathology towards several brain regions sending afferents to the ventral striatum such as the ipsilateral amygdala and substantia nigra, as well as the median layer of several neo- and paleo-cortical regions. These regions all contained numerous neuronal inclusions (LB-like perikaryal aggregates, NIIs, and LNs) that appeared positive using anti-phosphoS129 α-Syn antibodies (pSyn#64 and EP1536Y) and also, but with a lesser sensitivity, using the conformation-dependent antibody syn-303 [36] (not shown). In agreement with the reference reports using PFFs in vivo [37,38] and with our own observations using 1B fibrils [20], we found that none of these cytopathological inclusions could be detected in control animals, and that they corresponded to de novo aggregates resulting from the seeding/templating of endogenous mouse α-Syn and not to the migration/compaction of the PFF seeds. Indeed, we found that the pSyn-positive aggregates did not contain human fibrils that could be revealed with the human α-Syn-specific antibodies MJFR1 or LB-509 (not shown). 

**Figure 3:**
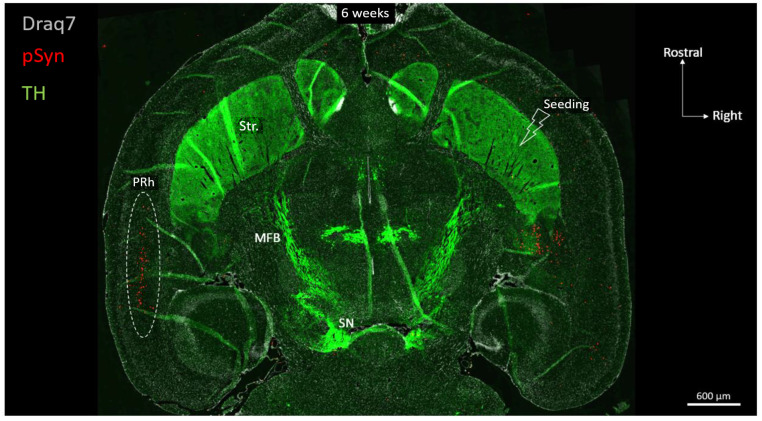
Horizontal topography of Tyrosine Hydroxylase (green, TH), α-Syn pathology (red, pSyn), and cell nuclei (white, Draq7) 6 weeks after seeding with 1B fibrils into the right striatum of a wild-type mouse (10 µm full brain section). SN: Substantia nigra; MFB: median forebrain bundle; Str.: ventral striatum (nucleus accumbens core and shell); PRh: perirhinal cortex.

Figure 3 shows a full brain semi-horizontal paraffin section of such an animal in which the full span of the nigrostriatal tract can be seen in green (anti-Tyrosine hydroxylase antibody, TH). 

The level of the section is ventral enough to encompass the ventral striatum (nucleus accumbens core and shell), the anterior commissure limbs, the median forebrain bundle, and the ventral part of the substantia nigra. The pSyn-positive aggregates (pSyn#64 antibody) appear in red, and with this level of zoom one can clearly perceive the invasion of the ipsilateral amygdala and entorhinal cortex, but also and most strikingly, of the median layer of the contralateral perirhinal cortex (layer V). The quick and massive spread of the α-Syn pathology to contralateral somas of cortical neurons is due to the coincidence of 2 constraints: an anatomical one, and a functional one. 

**Figure 4:**
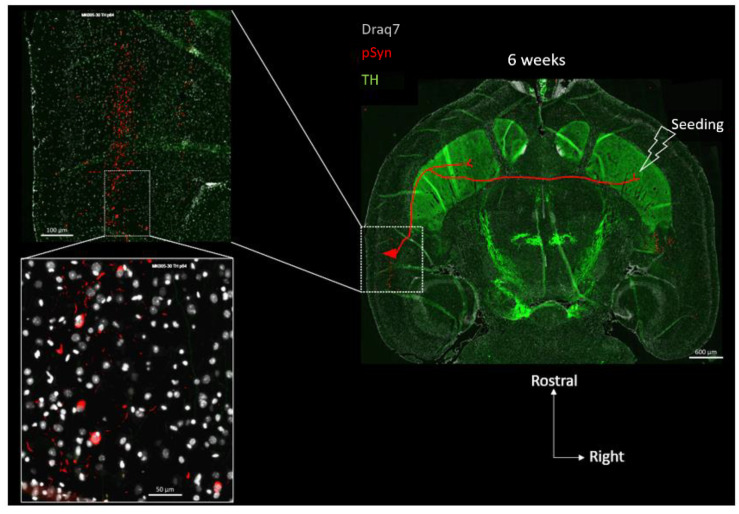
Concatenated zoomed views of the α-Syn pathology in the contralateral perirhinal cortex. The section is the same as in Figure 3 with Tyrosine Hydroxylase in green (TH), α-Syn pathology in red (pSyn, pSyn#64 antibody), and cell nuclei in white (Draq7) 6 weeks after seeding with 1B fibrils (10 µm full brain section). The most zoomed view shows five neuronal somas with Lewy-body-like aggregates, and numerous surrounding Lewy neurites. Red drawing in the topographic view: schematic sketch of a perirhinal cortico-striatal neuron with a contralateral projection passing through the anterior commissure.

Anatomically, the perirhinal cortex contains crossing cortico-striatal neurons that send their projections to the other hemisphere in which they establish synaptic contacts in the ventral striatum (Figure 4) [39,40,41,42,43]. The somas of these crossing perirhinal cortico-striatal neurons are present in the same layer V as the ones of the pyramidal neurons (Figure 4, see the two concatenated zoomed views), but send their axons in the posterior limb of the anterior commissure to reach the other hemisphere [41,42,43]. Regarding the functional constraint, it is well known that in vivo PFFs preferentially seed α-Syn pathology at the level of terminal synapses, and that the aggregation process then spreads retrogradely along the axons to eventually reach the distant somata [44,45] (reviewed in [46]). If this holds true in our experimental conditions, then the combination of both these anatomical and functional constraints implies that pSyn-positive aggregates corresponding to LNs should also be observed in axons transiting through the posterior limb of the anterior commissure. 

**Figure 5:**
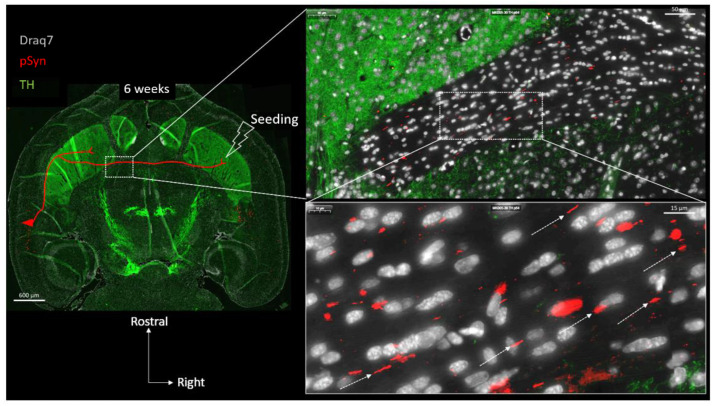
Concatenated zoomed views of the α-Syn pathology in the posterior limb of the anterior commissure. The section is the same as in Figure 3 and Figure 4 with Tyrosine Hydroxylase in green, (TH) α-Syn pathology in red (pSyn, pSyn#64 antibody), and cell nuclei in white (Draq7) 6 weeks after seeding with 1B fibrils (10 µm full brain section). The zoomed view shows numerous Lewy Neurites with a parallel course (dotted arrows) corresponding to the commissural axis. Red drawing in the topographic view: schematic sketch of a perirhinal cortico-striatal neuron with a contralateral projection passing through the anterior commissure.

Figure 5 shows two concatenated zoomed views of the posterior limb of the anterior commissure (in the same section as the one shown in Figure 3 and Figure 4), contralateral to the seeding side. 

In agreement with the previous considerations, many pSyn-positive LNs are indeed detected, delineating the parallel trajectories of the axons traveling in this anatomical structure (Supplementary Figure S2 confirms that the latter commissural LNs represent pathological segments of crossing Neurofilament-positive axons). This region is of particular interest because it can be considered as a “connecting wire” containing no neuronal somata or dendrites, but only traveling axons that are “insulated” by myelin sheaths [47] (Figure 1 and Figure 2). Thus, the vast majority of the cell nuclei that are detected in the anterior commissure correspond to interfascicular OLs (around 90%) with the remaining fraction of cell bodies corresponding to a few astrocytes and microglial cells [47] (Figure 1 and Figure 2). At 6 weeks, the LNs that collectively travel in parallel do not delineate any cell bodies.

**Figure 6:**
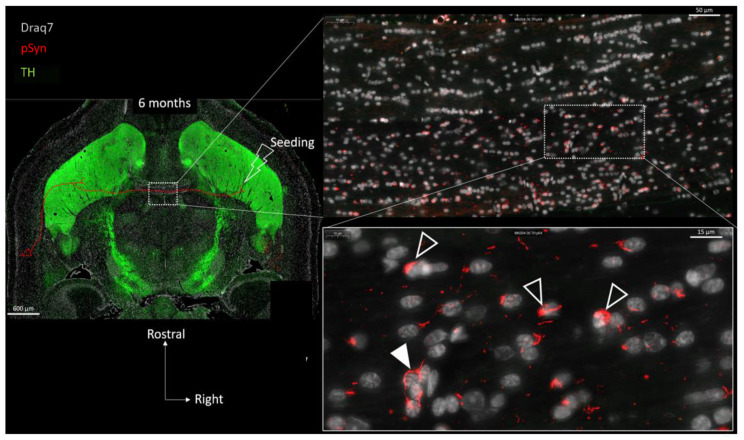
Concatenated zoomed views of the α-Syn pathology in the posterior limb of the anterior commissure 6 months after seeding with 1B fibrils (10 µm full brain section). Tyrosine Hydroxylase is in green (TH), α-Syn pathology in red (pSyn, pSyn#64 antibody), and cell nuclei in white (Draq7). The zoomed view shows numerous fragmented Lewy neurites no longer presenting an ensemble directionality or parallelism. Empty arrowheads point to curled-up Lewy neurites stuck to glial nuclei. Plain arrowhead shows Lewy neurite squeezed in-between several glial cell bodies. Drawing in the topographic view: schematic sketch of a perirhinal cortico-striatal neuron with a contralateral projection passing through the anterior commissure.

Figure 6 shows the drastic change that can be observed after 6 months compared to 6 weeks: the many LNs that are detected are no longer parallel, they appear fragmented, contorted, and curled up (empty arrowheads). They no longer delineate the interhemispheric course of axons, and can even be perpendicular to it, squeezed in-between two consecutive OLs (plain arrowhead). Interestingly, the most curled-up LNs appear stuck to the glial nuclei (presumably OLs) and/or wrap them, suggestive of a very close interaction or even of a glial internalization of the aggregate. 

**Figure 7:**
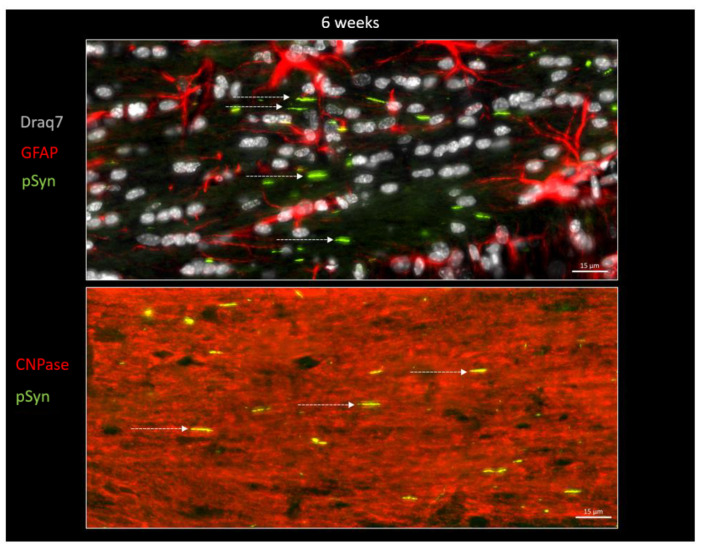
α-Syn pathology (pSyn, EP1536Y antibody, green) observable in the posterior limb of the anterior commissure in two other animals, 6 weeks after seeding with 1B fibrils (10 µm brain section). Upper panel: cell nuclei in white (Draq7), astrocytes in red (GFAP). Lower panel: myelin sheaths are in red (CNPase). Note the ensemble parallel course of Lewy neurites (dotted arrows).

**Figure 8:**
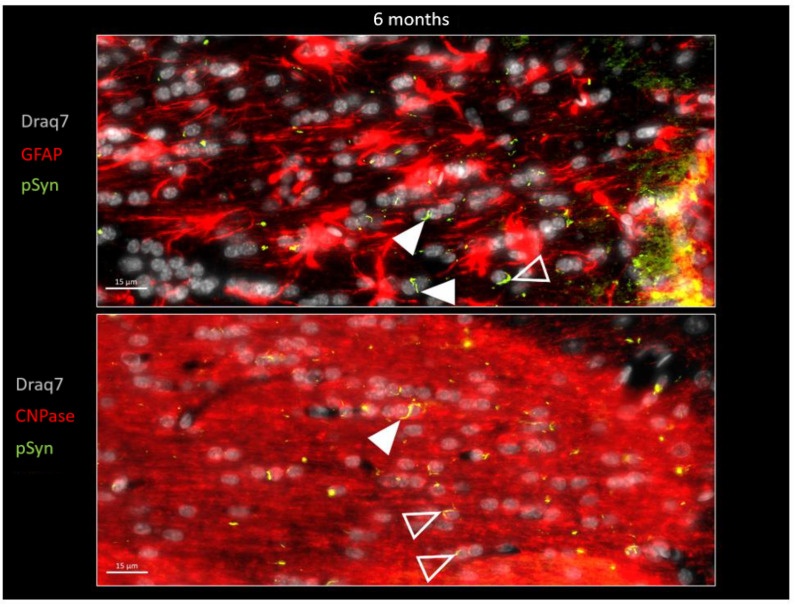
α-Syn pathology (pSyn, EP1536Y antibody, green) observable in the posterior limb of the anterior commissure in two other animals, 6 months after seeding with 1B fibrils (10 µm brain section). Upper panel: astrocytes are in red (GFAP). Lower panel: myelin sheaths are in red (CNPase), cell nuclei in white (Draq7). Note the loss of parallelism and the fragmented Lewy Neurites. Plain arrowheads: squeezed Lewy Neurites. Empty arrowheads: curled-up Lewy Neurites stuck to nuclei that seem to be internalized.

In order to further document this phenomenon, immunofluorescence detection of GFAP and of CNPase was also carried out on other brain sections in order to spot, respectively, the astrocytes and the myelin sheaths together with the LNs at both 6 weeks and 6 months (Figure 7 and Figure 8). Figure 7 again shows the ensemble parallel course of commissural LNs observable at 6 weeks (pSyn positive), corresponding to the axonal route highlighted by the myelin sheaths (CNPase positive). At this stage, the LNs are not delineating cell bodies, and are not correlated with the position of astrocytes (GFAP). Figure 8 confirms the tremendous change observed at 6 months, with the loss of parallel organization, the fragmentation and the curl-up of LNs (pSyn), with a few “perpendicular” LNs squeezed in-between perifascicular OLs (plain arrowheads), and certain images compatible with an internalization by OLs with a cell body delineated by CNPase (empty arrowheads). At this stage also, the distribution of fragmented/curled-up LNs does not appear to be correlated with the position of astrocytes (GFAP).

**Figure 9:**
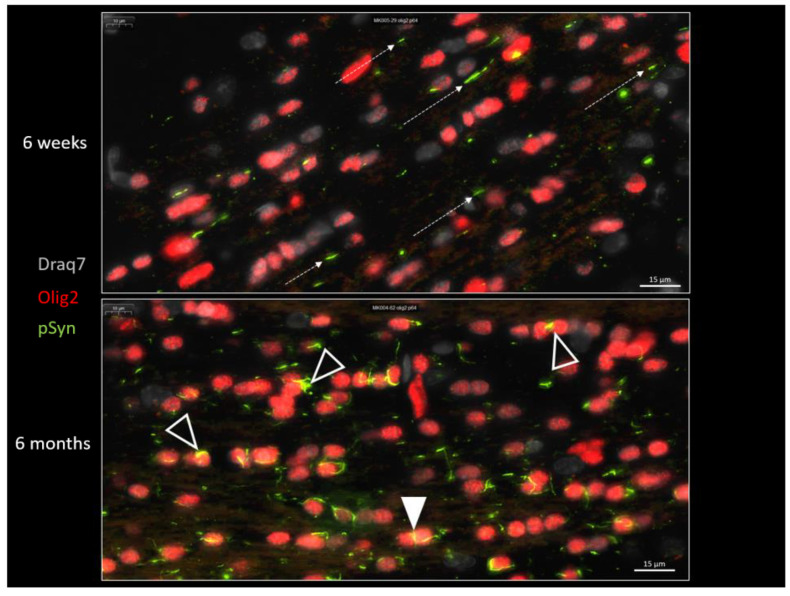
α-Syn pathology (pSyn, pSyn#64 antibody, green) observable in the posterior limb of the anterior commissure, 6 weeks (upper panel) or 6 months (lower panel) after seeding with 1B fibrils (10 µm brain section). Note the loss of parallelism (dotted arrows) and the fragmented Lewy Neurites at 6 months. Plain arrowhead: squeezed Lewy Neurite. Empty arrowheads: curled-up Lewy Neurites stuck to oligodendrocyte nuclei that seem to be internalized.

Figure 9 shows the distribution of LNs (pSyn in green) and of the OL nuclear marker Olig2 (in red) at 6 weeks and 6 months after seeding, in the posterior limb of anterior commissure. This figure confirms the transformation of the parallel LN ensemble into fragmented and curled-up LNs, squeezed (plain arrowhead) or delineating/wrapping the OL nuclei, and eventually forming aggregates with an appearance compatible with a process of internalization into OLs (empty arrowheads). 

**Figure 10:**
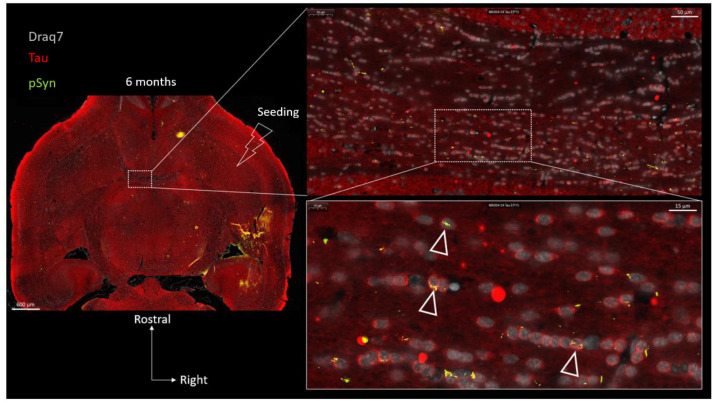
Concatenated zoomed views of the α-Syn pathology in the posterior limb of the anterior commissure 6 months after seeding with 1B fibrils (10 µm full brain section). Tau is in red, (Tau) α-Syn pathology in green (pSyn, EP1536Y antibody), and cell nuclei in white (Draq7). The zoomed view shows numerous fragmented Lewy Neurites no longer presenting an ensemble directionality or parallelism. Empty arrowheads point to curled-up Lewy Neurites internalized into Tau-positive oligodendrocytes forming GCIs and GNIs.

**Figure 11:**
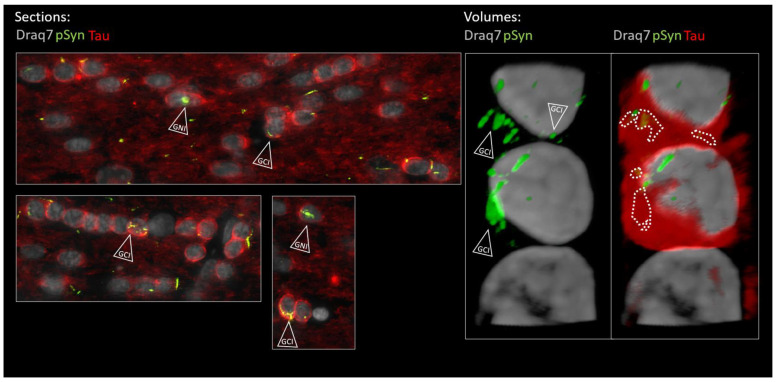
Enlarged 2D (section of Figure 10) and 3D (volume) views of the oligodendroglial α-Syn pathology in the posterior limb of the anterior commissure 6 months after seeding with 1B fibrils (10 µm full brain section). Tau is in red (Tau), α-Syn pathology in green (pSyn, EP1536Y antibody), and cell nuclei in white (Draq7). The two lower panels in the “Sections” are further zoomed views of Figure 10. Empty arrowheads point to curled-up LNs internalized into Tau-positive oligodendrocytes forming GCIs and GNIs. The dotted contours delineate the α-Syn pathology that was internalized and which is thus masked by cytoplasmic Tau in the volume view. The volume was reconstructed from a z-stack acquired using confocal microscopy. See the corresponding animation in the Supplementary Movie. Note the presence of a halved nucleus at the base of the strand that belongs to an astrocyte (Tau negative, scattered chromatin). (Scale bars omitted, OL nucleus diameter ≈ 5 µm).

In order to investigate the possible localization of the latter aggregates inside the cytoplasmic compartment of the OLs, we looked for a cytoplasmic OL marker that would not decorate myelin sheaths such as CNPase, MBP, or MOG (not shown) to avoid “constructed images” due to the myelin-rich environment. To our surprise, we incidentally observed that the protein Tau, which is yet acknowledged as a bona fide marker of neuronal axons, was barely decorating the axons traveling in the anterior commissure (irrespective of the presence of LNs) compared to cortical or striatal regions for instance (Figure 10, topographic section view). Instead, many interfascicular OLs exhibited the clearcut presence of Tau in their cell bodies, more precisely in their perikarya (zoomed view in Figure 10 and Figure 11). The presence of Tau in the OL cell bodies, which we initially considered as an odd observation, was instead confirmed by several previous reports in which series of anti-Tau antibodies were screened [23,24,48,49] and single cell transcriptomics analyses were conducted [50], all leading to a similar conclusion: mature OLs do express Tau to very significant levels. For a comprehensive review see [23]. We thus took advantage of the clearcut cytoplasmic Tau signal in the commissural OLs showing up against a very low Tau background in the commissural axons (see also Supplementary Figure S3) to address the possible internalization of curled-up LNs into the OL cell bodies at 6 months (Figure 10 and Figure 11). Figure 10 shows concatenated zoomed views of Tau (red) and pSyn (green) distributions starting from the topographical view of the full brain section, to culminate in a close-up view of the posterior limb of the anterior commissure. In agreement with the previous observations, the commissural LNs (pSyn) are no longer parallel at this stage but are fragmented and curled up. Tau staining, which reveals the cytoplasm of the commissural OLs in red, makes it clear that most if not all of the curled-up LNs found closely associated with the cell body/nucleus of OLs are in fact properly internalized inside the OL cell bodies (a few examples are shown by empty arrows). This observation was confirmed using the OL cell body marker Quaking-7 [22] (Supplementary Figures S4 and S5). Figure 11 shows further zoomed views of the commissural OLs from the section shown in Figure 10 that allow one to distinguish the two types of aggregates formed in OLs by the internalized LNs: Glial Cytoplasmic Inclusions that remain confined to the cytoplasm (GCI-labeled arrows), and also Glial Nuclear Inclusions (GNI-labeled arrows), which are also cytopathological lesions that are pathognomonic of MSA [51].

## 4. Discussion

**Figure 12:**
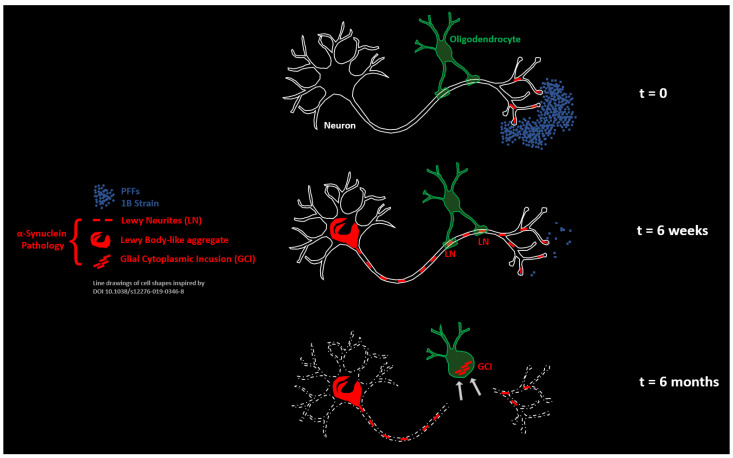
Pruning of LNs by commissural oligodendrocytes is at the origin of GCIs in mice treated with the human α-Syn fibril strain 1B. Schematic model.

In this study we exploited the specific anatomy of the inter-hemispheric cortico-striatal connections evidenced by a horizontal brain sectioning histological approach. This allowed us to put the anterior commissure under scrutiny during the interhemispheric axonal retrograde spread of an experimental α-Syn pathology seeded by the human α-Syn fibril strain 1B in mice. Using two time points we could observe the formation and the morphological transformation of LNs corresponding to segments of interhemispheric axons filled with neo-formed α-Syn aggregates. In the anterior commissure, these axons are myelinated and surrounded by a dense population of mature OLs, and we observed that the linear LNs that initially formed during the onset of the α-Syn pathology were conforming to the parallel course of the axons traversing this anatomical structure in the horizontal sectional plane. After 6 months, however, the picture radically changed: while a lot of LNs were still present in the commissure, they no longer showed a parallel organization. Instead they were contorted, fragmented, squeezed in-between OLs arranged in interfascicular strands, and stuck to OLs as curled-up masses. These latter forms were eventually internalized in OLs forming GCIs and GNIs. Our observations suggest the existence of an aggregate import mechanism consisting of the pruning and the engulfment of diseased axonal segments (LNs) by OLs (Figure 12). It is worth noting that axonal pruning by OL precursor cells has been described during brain development [52,53], as well as in the visual cortex of adult mice [54]. Reactivation of this mechanism in mature OLs in the context of an axonal α-Syn pathology is a particularly interesting possibility that could explain how GCIs are formed during MSA. 

This mechanism provides an answer to the enigmatic appearance of α-Syn inclusions in cells not expressing α-Syn, in which the seeding of an α-Syn pathology should otherwise be precluded (Figure 1) (also discussed in [30]). It is also in agreement with the observation that insoluble α-Syn aggregates from MSA patients with GCIs display a prominent neuronal proteomic fingerprint which is suggestive of an import of GCIs from neurons as preassembled inclusions [29]. It is interesting to note that the α-Syn fibril strain 1B, which is capable of seeding in vivo the formation of MSA-specific inclusions types NIIs [34], GCIs, and GNIs (present work), is also a strain capable of causing a distinctively florid neuronal LN α-Syn pathology compared to other fibril strains [20]. From this point of view, it is thus tempting to speculate that strains specifically causing the prolonged persistence of α-Syn aggregates within axonal segments, i.e., of LNs, could favor axonal pruning and engulfment by OLs and thus be the cause of the appearance of GCIs. This speculation finds support in the recent demonstration of a particularly rich LN pathology in the transverse pontine fibers of MSA patients, combined with the presence of GCIs in the oligodendroglial cell bodies within those bundles and of NCIs within the neuronal perikarya of the pontine nuclei which give rise to those fibers [55]. 

Heuristically, we speculate that the extended remanence of axonal LNs caused by MSA α-Syn strains could favor their engulfment in OLs (thus forming GCIs and GNIs), while in PD and DLB, different α-Syn strains would cause the formation of axonal aggregates capable of a faster retrograde progression towards the neuronal somata, leaving no sufficient time to the OLs to react to the transiting aggregates. This would explain the absence of GCIs in PD and DLB. 

According to our proposal, the formation of GCIs by axonal pruning would thus be expected to originate in brain regions that are rich in myelinated axons, in other words, in the white matter. This is the case in MSA, in which a drastic degeneration of the white matter takes place with a loss of myelinated fibers without major change in the number of OLs [56]. Additionally, it is worth noting that (i) as early as 1989, Papp, Kahn, and Lantos described that the appearance of GCIs was concomitant with a widespread myelin loss associated with axonal degeneration [57], and that (ii) the white matter changes in MSA are prominent enough to be readily detected in patients during their lifetimes by employing digital tensor imaging [58,59]. Thus, beyond promoting the concept of GCIs as “imported LNs”, the process of axonal pruning we experimentally modeled here (schematized in Figure 12) could also turn out to be causal in the demise of the neurons bearing axonal α-Syn aggregates. This possibility and its relevance to the pathophysiology of MSA are under investigation.

## Figures and Tables

**Table 1:** Low ThT detectability of mouse and human α-Syn synthetic fibril strains related to MSA compared to reference human α-Syn fibrils, or fibrils templated using PD brain homogenates (BH). ThT A.U. value pairs from articles cited as DOIs.

	DOI	ThT Value for Equal Amounts of α-Syn Fibrils (Variable A.U.)	Fold Difference
**mouse α-Syn fibrils**	10.1016/j.celrep.2016.08.053	**40**	**3.75**
human α-Syn fibrils		150	
**human α-Syn ribbons**	10.1038/ncomms3575	**4**	**8.25**
human α-Syn fibrils (type 2)		33	
**MSA-templated α-Syn fibrils (BH)**	10.1038/s41586-020-1984-7	**480**	**12.7**
PD-templated α-Syn fibrils (BH)		6100	
**human** **α** **-Syn fibrils 1B**	10.3390/biom12030436	**2437**	**10.3**
human α-Syn fibrils (type 2)		25100	

## Data Availability

Data are available upon request.

## Supplementary Materials

The following supporting information can be downloaded at: https://www.mdpi.com/article/10.3390/biom13020269/s1, Supplementary Movie S1: 3D animation of the volume shown in Figure 11. For color codes see Figure 11 Legend.
