# Supplementary material for: Oligodendrocytes Prune Axons Containing α-Synuclein Aggregates In Vivo: Lewy Neurites as Precursors of Glial Cytoplasmic Inclusions in Multiple System Atrophy?
Source: Biomolecules. 2023 Feb 1;13(2):269. doi: 10.3390/biom13020269 (PMC9953613; doi:10.3390/biom13020269)
Supplement: Supplementary file 1 [file biomolecules-13-00269-s001.zip › biomolecules-2124592-supplementary/biomolecules-2124592-supplementary-revised.pdf]

Figure S1

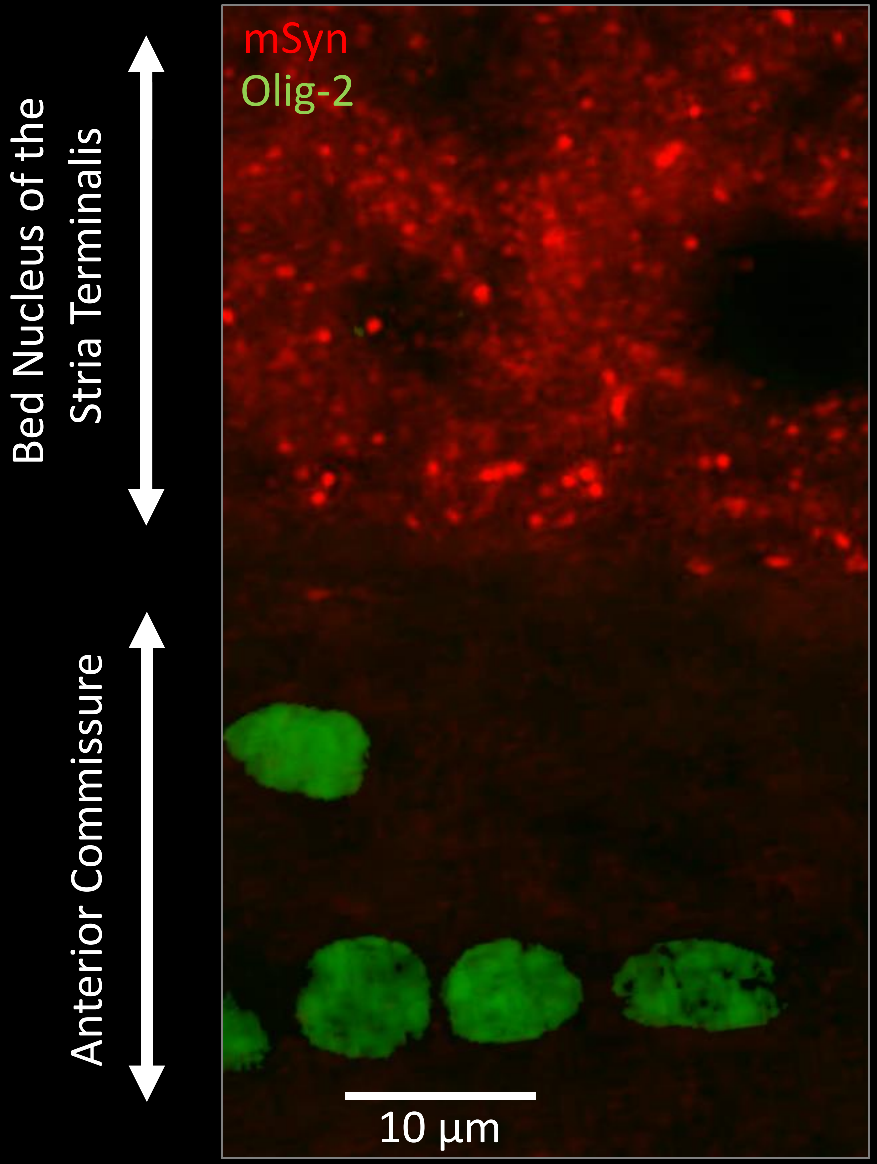

Fig. S1 Legend: Double immunofluorescence revealing interfascicular OLs (Olig-2, 211F1.1 antibody, green) and mouse α-Syn (mSyn, D37A6 antibody, red) at the level of the transition zone between the anterior commissure (white matter) and the bed nucleus of the stria terminalis (grey matter) of a control mouse. Horizontal brain section. Note the synaptic distribution of α-Syn in the grey matter (black voids surrounded by synapses correspond to neuronal somatas), and its absence in OLs and more generally, in the commissural comptartment.

Figure S2

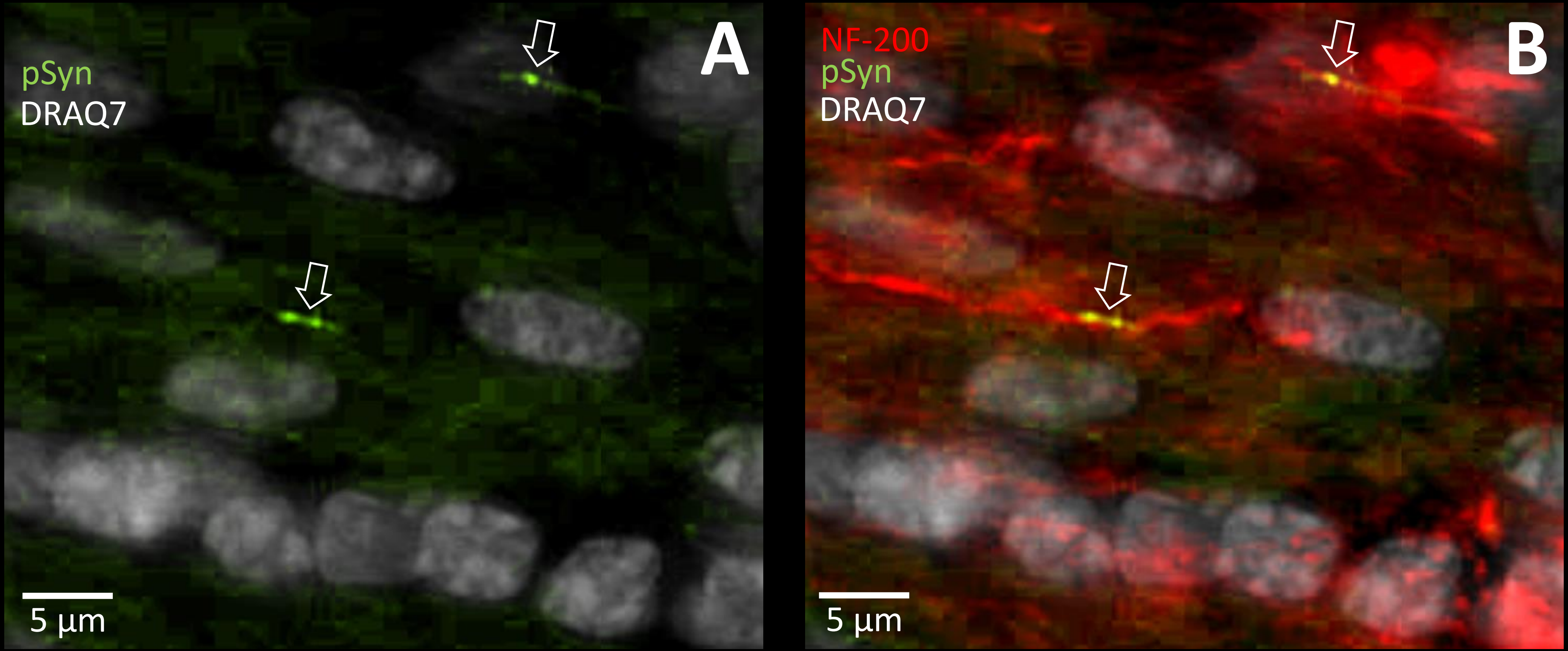

Fig. S2 Legend: Double immunofluorescence revealing Lewy neurites (pSyn, Green) and crossing axons (NF-200, red) at the level of the anterior commissure of a mouse 6 weeks after the intrastriatal injection of α-Syn PFFs. Horizontal brain section. Nuclei are counterstained with DRAQ7 (white). In A, only pSyn is shown, note the 2 Lewy neurites (empty arrows). In B, both NF-200 and pSyn are shown, evidencing the axonal nature of the Lewy neurites.

Figure S3

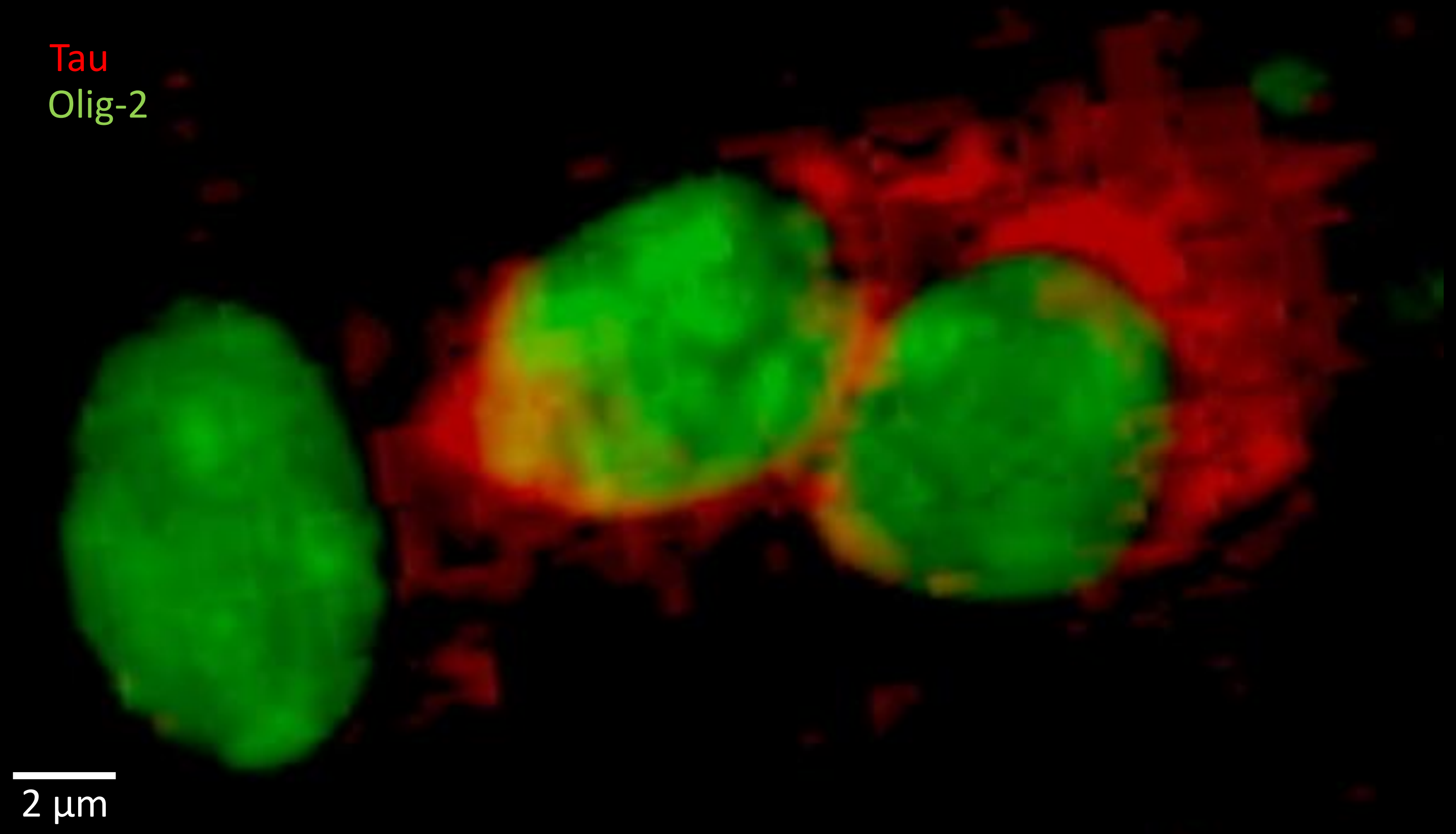

Fig. S3 Legend: Double immunofluorescence revealing the nuclei of 3 interfascicular OLs (Olig-2, Green) and of Tau (T47 antibody, red) in the anterior commissure of a control mouse. Horizontal brain section. In agreement with previous reports, Tau is most often present in the cytoplasm of OLs (2 out of 3 OLs in this image) reflecting the level of differentiation of the OLs.

Figure S4

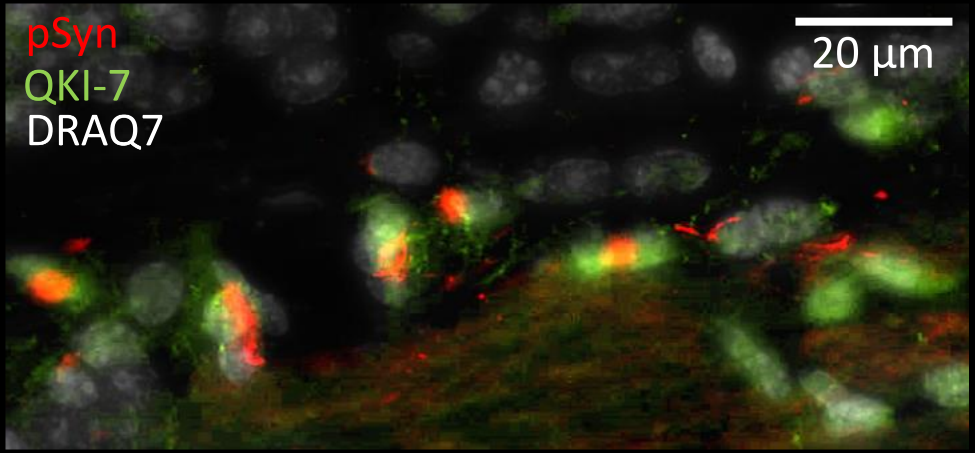

Fig. S4 Legend: Double immunofluorescence revealing pruned Lewy Neurites (pSyn, Red) and the cytoplasms of mature OLs (QKI-7, green) at the level of the anterior commissure of a mouse 6 months after the intrastriatal injection of α-Syn PFFs. Horizontal brain section. Nuclei are counterstained with DRAQ7 (white).

Figure S5

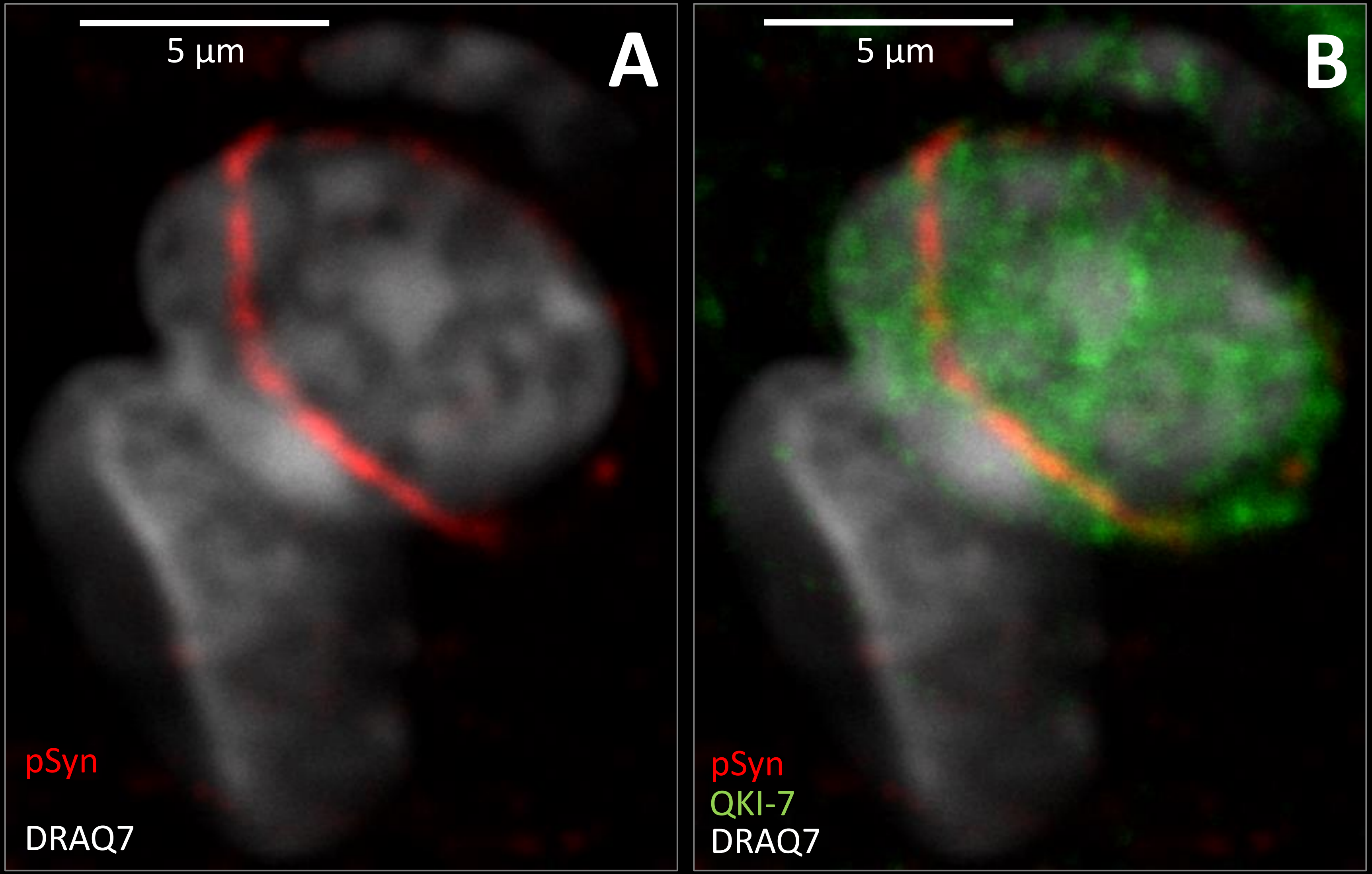

Fig. S5 Legend: Confocal imaging of a double immunofluorescence showing a mature OL (QKI-7, green) pruning a Lewy Neurite (pSyn, red) in the anterior commissure of a mouse 6 months after the intrastriatal injection of α-Syn PFFs. Horizontal brain section. Nuclei stained with DRAQ7 (white). In A, only pSyn is shown while in B, both pSyn and the mature OL marker QKI-7 are shown.
